# Quantifying the Residual Stiffness of Concrete Beams with Polymeric Reinforcement under Repeated Loads

**DOI:** 10.3390/polym15163393

**Published:** 2023-08-13

**Authors:** Haji Akbar Sultani, Aleksandr Sokolov, Arvydas Rimkus, Viktor Gribniak

**Affiliations:** Laboratory of Innovative Building Structures, Vilnius Gediminas Technical University (VILNIUS TECH), Sauletekio av. 11, LT-10223 Vilnius, Lithuania; haji-akbar.sultani@vilniustech.lt (H.A.S.); aleksandr.sokolov@vilniustech.lt (A.S.); arvydas.rimkus@vilniustech.lt (A.R.)

**Keywords:** reinforced concrete, fiber-reinforced polymer (FRP), bending test, analytical model, residual strength, repeated loads

## Abstract

Current technology development ensures a variety of advanced materials and options for reinforcing concrete structures. However, the absence of a uniform testing methodology complicates the quantification and comparative analysis of the mechanical performance of the composite systems. The repeated mechanical loads further complicate the issue. This research extends the recently developed residual stiffness assessment concept to the repeated loading case. It provides an engineer with a simplified testing layout and analytical model to quantify the residual flexural stiffness of standardized laboratory specimens subjected to repeated cycling loads. This model explicitly relates the particular moment and curvature values, requiring neither iterative calculations nor the load history. Thus, this feature allows residual stiffness quantification under repeated loading conditions, including complete reloading of the beam samples imitating the structural strengthening procedure; the proposed technique is equally efficient in quantifying the residual stiffness of the beam samples with any combinations of fiber-reinforced polymer (FRP) reinforcements, i.e., embedded bars, near-surface-mounted strips, and externally bonded sheets. This study employs 12 flexural elements with various reinforcement and loading layouts to illustrate the proposed methodology’s efficiency in quantifying the residual strength of the tension concrete, which estimates the efficiency of the reinforcement system. The explicit quantifying of the residual resistance of the FRP reinforcement systems under repeated load cycles describes the essential novelty of this work.

## 1. Introduction

### 1.1. Literature Review

Technology development provides various materials for reinforcing and strengthening concrete structures [1,2]. Fiber-reinforced polymers (FRPs) are a promising steel alternative because they are high-strength, lightweight, immune to corrosion, and electromagnetically transparent [3,4,5,6]. Still, the relatively low resistance to ultraviolet radiation, elevated temperatures, and humidity reduce the mechanical performance of FRP materials with time [7,8,9]; the cycling loads complicate the issue, reducing the mechanical resistance of the reinforcement systems even more [10,11]. Regarding FRP bars, most experimental works consider the pull-out behavior under cyclic loads, e.g., Mohamed et al. [12], Kim and Lee [13], Liu X. et al. [14], Shen et al. [15], Xiao et al. [16], Shi et al. [17], and Pan et al. [18], which could transform into the structural analysis. However, this “transformation” describes a non-trivial problem mainly related to the local bond treatment. For instance, in the tests [12,13,14,15,16,17,18], the bond length varied from 60 mm to 130 mm for the bar diameters 10 mm, 12 mm, 13 mm, and 14 mm. Notwithstanding the apparent efficiency in estimating the bond performance, these test results are unrepresentative of structural elements, which do not face such severe bond deformations in the service conditions [19]. Still, these tests isolate a single bar behavior, which is unrealistic in most structural applications.

Various FRP materials, i.e., embedded bars, near-surface mounted (NSM) strips, and externally bonded reinforcement (EBR) sheets, exhibit diverse mechanical performances when reinforcing composite systems. The literature has extensively documented these performances [20]. Studies by Rimkus et al. [21] and Gribniak et al. [22] have shown that combining FRPs with steel reinforcement effectively addresses engineering challenges. Anas et al. [23] investigated the structural performance of square slab specimens reinforced with different types of FRPs, and they found that samples with CFRP bars exhibited exceptional impact resistance. Using a four-point bending test, Yuan et al. [24] examined the flexural behavior of reinforced concrete (RC) beams strengthened with GFRP tubes. The results demonstrated that the strengthening solution improved the composite element’s flexural strength and bending stiffness under investigation. Farahi et al. [25] conducted three-point bending tests on concrete beams reinforced with composite materials, demonstrating such beams’ ductility and energy dissipation potential under monotonic and repeated loading. However, the variety of the specimen shapes and loading conditions does not allow comparing the test outcomes to optimize the reinforcement parameters. For instance, Godat et al. [26] concluded that the effective axial strains in composite reinforcement are higher in smaller specimens, while larger samples exhibit lower deformation in the FRP component. Therefore, a unified testing procedure is necessary to compare different reinforcement systems adequately.

Concrete structures often experience cyclic rather than static loading in practical engineering applications. Li et al. [27] investigated the influence of concrete strength and reinforcement parameters on the flexural behavior and ductility of concrete beams reinforced with basalt FRP bars under repeated loading. The experimental results showed a reduction in the peak load of the beams with an increase in the number of loading cycles at a constant deflection. Previous research by Kargaran and Kheyroddin [28] and Sultani et al. [29] supports the notion that repeated mechanical loading introduces complexities in the behavior of structural composites. Therefore, it is crucial to examine the structural performance of concrete beams reinforced with FRP bars under cyclic loading to advance the development of composite reinforcement in concrete structures.

An alternative approach to studying the flexural behavior of RC specimens under various loading conditions is using the acoustic emission method. Mat Saliah and Md Nor [30] extensively investigated this method in the context of assessing the structural integrity of concrete beams reinforced with composite materials. This technique evaluates microscopic damage within concrete elements caused by different external loading conditions. However, its predictive capability for the structural performance of the investigated specimens is limited. Another approach is to predict flexural behavior using numerical simulations. For example, Sun et al. [31] numerically studied the static and dynamic performances of a concrete beam reinforced with FRP bars using a simplified spectral model. This numerical approach shows promise for dynamic analysis by considering changes in dynamic characteristics. However, it is not suitable for static analysis.

The absence of a standardized methodology for quantifying reinforcement efficiency in composite systems subjected to repeated loads motivated this study. Several studies have examined the behavior of RC beams with composite reinforcement under repeated loading. For instance, Fathuldeen and Qissab [32] investigated the mechanical performance of RC beams strengthened with a CFRP NSM system under low cycle repeated loads. This study proved the efficiency of the hybrid reinforcement systems in resisting repeated load, highlighting the optimization importance of the steel and CFRP reinforcement proportions. These outcomes align with the monotonic loading test results and conclusions [21]. Zhu et al. [33] investigated the fiber effect in improving the mechanical resistance of RC beams with steel and FRP bars under repeated loads. The tests revealed the fiber efficiency in improving the concrete’s tensile resistance and compression ductility. These results also agree with the monotonic test outcomes [34]. The research in [33] also observed a substantial decrease in the flexural strength (up to 90%) of the beams with FRP reinforcement after increasing the load intensity. Song et al. [35] identified the adverse effect of cycling loads on the ultimate resistance of CFRP bars used in concrete frames in combination with steel reinforcement. However, the repeated loading effect on the residual stiffness of RC elements, which is predominant for normal (service) structural conditions, lacks adequate attention in the literature.

### 1.2. The Proposed Standardized Analysis Concept

Unlike the structural analysis design, the proposed procedure focuses on the mechanical performance of composite materials, particularly reinforcement systems. As described in the previous section, recent achievements in engineering, developing advanced fibers and reinforcement materials (steel and non-metallic), and other reinforcement solutions caused problems in setting an efficient solution for a particular situation. Furthermore, the combination of fibers and continuous reinforcement (either embedded bars, near-surface-mounted strips, or externally bonded laminates) in hybrid systems (e.g., [10,11]) complicates the reinforcement efficiency analysis.

As mentioned in Section 1.1 and reported in references [4,36], the absence of a uniform testing methodology complicates the comparative analysis of the efficiency of alternative materials and reinforcement layouts reported in the literature. Furthermore, the analysis of full-scale objects is possible but relevant only for the verification of particular solutions. However, it is unpractically expensive for studies involving a variety of potentially feasible solutions.

Therefore, Gribniak et al. [20,22] developed a “standardized” testing procedure to ensure the comparative analysis and simulation of various possible loading situations to select several feasible solutions for further investigation, e.g., full-scale tests. The monotonic loading tests verified the adequacy and reliability of the developed testing layout, chosen geometry of the sample, and analytical model, which explicitly quantifies the residual stiffness of the reinforced specimen expressed in terms of equivalent stresses in the tension concrete. Reaching zero, the latter value corresponds to the total loss of the bonding performance of the reinforcement, also known as the composite action or tension-stiffening effect [37]. Thus, the term “standardized” describes the peculiar geometry of the laboratory samples and testing methodology developed to satisfy the simplified modeling assumption (i.e., the rectangular distribution of stresses in the concrete in tension). In other words, the “exact” average stress–average strain tension-stiffening diagram [38] has a rectangular shape close to the rectangular approximation assumed in this study. Thus, the equivalent stress–strain relationships do not represent analytical material models but a quantitative estimate of the tension-stiffening effect.

At the same time, the comparative analysis of the equivalent stress–strain relationships of alternative reinforcement systems can estimate the improvement of the reference solution. For instance, Gribniak and Sokolov [36] evaluated the fiber-bridging effect in the presence of bar reinforcement by comparing the plain concrete and fiber-reinforced concrete “standardized” samples. The obtained difference between equivalent stress–strain relationships determined the material model suitable for the numerical simulation of the fiber-bridging effect in full-scale beams. The considered small-size samples can also verify finite element models representing a peculiar geometry case [39]. However, notwithstanding the apparent benefits of laboratory testing, reducing the length of the “standardized” samples can cause reinforcement anchorage problems [17], which require particular care in designing the test programs.

Jakubovskis et al. [40] transformed this approach to quantify the bacterial healing effect in RC samples. It also can be extended to determining the residual strength of the fiber-reinforced concrete with bar reinforcement [36]. Furthermore, this testing procedure ensures the residual stiffness analysis of elements subjected to temperature (including an open environment), ultraviolet radiation, and long-term (creep) effects [41]. In other words, the proposed tool helps quantify the reinforced material performance in composite systems. The term “composite” determines concrete with various combinations of reinforcements (e.g., steel and non-metallic fibers, embedded bars, near-surface-mounted strips, and externally bonded reinforcement in different combinations).

This study adapts the proposed testing layout and the simplified analytical model to analyze the residual flexural stiffness of the laboratory specimens subjected to repeated mechanical loads. It employs the concept of the equivalent residual stresses acting on concrete in tension to measure the structural performance of alternative composite reinforcement systems and their variation with the load cycles of various intensities. This analytical model explicitly relates the bending moment and curvature values, quantifying the equivalent stresses acting in the concrete under the assumption of the rectangular stress distribution. This solution requires neither iterative calculations nor the load history definition.

At the same time, the simplified analytical model [22] does not define concrete’s constitutive law in its traditional sense. The simplified nature of the model only ensures approximating the equivalent stresses with a sufficient degree of accuracy—from the analysis [22], the average approximation error regarding the exact solution [38] does not exceed 7%. However, the latter inverse analysis procedure is inapplicable to the specimens under cycled loads, making the proposed simplified analysis concept irreplaceable for analyzing the repeated load effects. This experimental study employs the flexural test results of 12 “standardized” beam samples with different arrangements and combinations of FRP reinforcement. This manuscript provides only an illustrative example of the proposed analysis procedure and tends to cover only some possible structural situations and loading conditions. The explicit quantifying of the residual resistance of the composite reinforcement systems under repeated load cycles describes this study’s essential novelty.

## 2. Testing Method and Analytical Model

**Figure 1:**
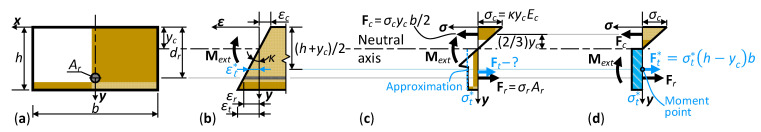
Analytical model of the stress distribution in the pure-bending zone [22]: (**a**) a transformed cross-section; (**b**) the bending-induced strain distribution along the cross-section height; (**c**) the corresponding stress distribution; (**d**) the equivalent stress approximation in the tension concrete zone. Note: the blue color shows the approximation procedure.

The explicit nature of the analytical expressions, which do not require any additional modifications regarding the previous publications [20,22,29], ensures the residual stiffness analysis of elements facing the load repetitions. Therefore, Section 2.1 briefly describes the simplified analytical model (Figure 1); reference [22] provides further explanations and the model verification results. Still, the unloading repetitions generate residual (permanent) deformations, which are mandatory for the proposed stiffness analysis procedure, as Section 2.2 discusses in detail. Section 2.3 describes the experimental campaign, illustrating the analysis technique.

### 2.1. Analytical Model

The model (Figure 1) considers the transformed reinforcement approach, which combines different materials in one component (Figure 1a). The following equations define the transformed reinforcement characteristics:

(1) $$\begin{array}{c} d_{r}=\sum_{i=1}^{n}E_{i}A_{i}d_{i}/\sum_{i=1}^{n}E_{i}A_{i},\;A_{r}=\frac{1}{E_{r}}\sum_{i=1}^{n}E_{i}A_{i},\;E_{r}=E_{1}, \end{array}$$

where *d_r_* is the effective depth; *A_r_* is the cross-section area; *E_r_* is the modulus of elasticity; *n* is the number of the different reinforcement parts; *E_i_*, *A_i_*, and *d_i_* are the modulus of elasticity, cross-section area, and effective depth of the *i*-th reinforcement component.

The proposed analytical model uses the following assumptions:

- The strain distribution follows the Euler–Bernoulli hypothesis (Figure 1b). Numerous literature sources proved the adequacy of this assumption for RC members (e.g., [42,43]).
- The smeared crack model describes the stress–strain behavior of the tension concrete (Figure 1b,c). Various literature examples (e.g., [37,38,43]) proved the correctness of this modeling concept.
- Idealized elastic material laws define the mechanical behavior of the reinforcement and the compressed concrete. This modeling approach substantially simplifies the mathematical expressions and ensures a straightforward solution.
- The rectangular distribution of the tensile stresses in concrete defines the equivalent stress $\sigma_{t}^{*}$ (Figure 1d). This center simplification ensures formulating the exact relationship between the bending moment and curvature and avoiding iterative solutions.

The latter two assumptions allow quantifying the stiffness of the beams subjected to repeated loads with the solution expressed in terms of the equivalent tensile stresses. The following equilibrium equations of internal forces and bending moments for the centroid of the equivalent stress diagram (Figure 1d) define the analytical model:

(2) $$\begin{array}{cc} F_{t}^{*}+F_{r}-F_{c}=0; & F_{r}\left(d_{r}-\frac{h+y_{c}}{2}\right)+F_{c}\left(\frac{h+y_{c}}{2}-\frac{y_{c}}{3}\right)-M_{ext}=0, \end{array}$$

where $F_{t}^{*}$ is the equivalent resultant force in the tensile concrete; **F***_r_* and **F***_c_* are the internal forces acting on the tensile reinforcement and the compressed concrete; *d_r_* is the efficient depth of the transformed reinforcement (Equation 1); *h* and *y_c_* are the height and gravity center coordinate of the cross-section in Figure 1. The model also allows the efficient depth to exceed the cross-section height (i.e., the *d_r_* > *h* condition is acceptable).

The above equation system relates the internal forces and stresses acting on the cross-section, employing the strain compatibility condition (Figure 1b). Reference [22] defines the intermediate solution steps; the final equation describes the third-order polynomial of the neutral axis coordinate *y_c_* for the particular curvature *κ* and bending moment **M***_ext_*:

(3a) $$\sum_{i=0}^{3}K_{i}y_{c}^{i}=0,$$

with coefficients

(3b) $$K_{3}=\frac{E_{c}b}{6E_{r}A_{r}},\;K_{2}=1+\frac{E_{c}bh}{2E_{r}A_{r}},\;K_{1}=h-3d_{r},\;K_{0}=2d_{r}^{2}-hd_{r}-\frac{2M_{ext}}{\kappa E_{r}A_{r}},$$

where *E_c_* is the modulus of elasticity of concrete; *h* and *b* are the height and width of the cross-section in Figure 1; *κ* is the average curvature of the pure-bending zone subjected to the external bending moment **M***_ext_*; Equation 1 describes the remaining parameters.

The above polynomial has three roots, and the [0 < *y_c_* ≤ *h*] condition describes the neutral axis position:

(4) $$\begin{array}{cc} y_{c}=\frac{2\bar{K}}{3K_{3}}\cos\left(\frac{1}{3}\cos^{-1}\left[-\frac{13.5K_{3}^{2}K_{0}-4.5K_{3}K_{2}K_{1}+K_{2}^{3}}{{\bar{K}}^{3}}\right]\right)-\frac{K_{2}}{3K_{3}}, & \bar{K}=\sqrt{K_{2}^{2}-3K_{3}K_{1}} \end{array}.$$

The following explicit formulas define the equivalent stress (Figure 1d) and average strain of the concrete in tension (Figure 1b):

(5) $$\begin{array}{cc} \sigma_{t}^{*}=\kappa \frac{y_{c}^{2}bE_{c}-2\left(d_{r}-y_{c}\right)E_{r}A_{r}}{2\left(h-y_{c}\right)b}, & \varepsilon_{t}^{*}=0.5\kappa \left(h-y_{c}\right) \end{array}.$$

The above expressions solve the residual strength problem independently of the loading conditions, explicitly relating the equivalent stress $\sigma_{t}^{*}$ with the bending moment **M***_ext_*, average curvature in the pure-bending zone *κ*, cross-section geometry (Figure 1a), and material parameters discussed in this section above. Thus, the proposed analytical model becomes applicable for quantifying the residual mechanical performance of the composite reinforcement system (expressed in terms of the equivalent stress $\sigma_{t}^{*}$ and equivalent strain $\varepsilon_{t}^{*}$ relationship) under repeated loading conditions considered in this work.

### 2.2. Sample Geometry and Testing Layout

**Figure 2:**
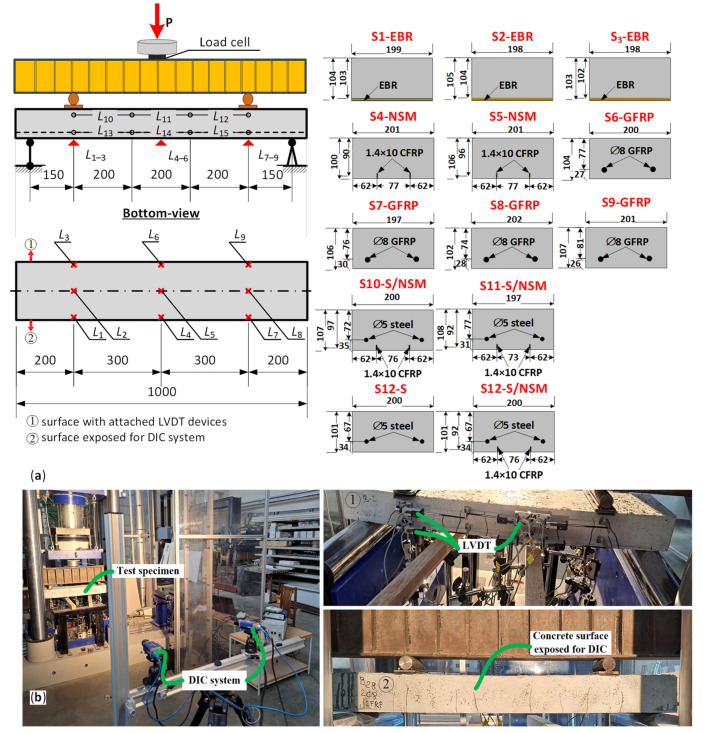
Test specimens (dimensions are in mm): (**a**) loading scheme; (**b**) experimental view.

Gribniak et al. [20,22] and Sultani et al. [29] established the geometry and the testing layout of the bending samples, following the formation of multiple cracks in a relatively small laboratory sample and thus reducing the discrete cracking effect on the curvature estimation result. Therefore, this study considers the 1000 mm slab-shaped beams loaded in the four-point scheme, as Figure 2a shows. The 200 × 100 mm cross-section was reinforced with near-surface mounted (NSM) strips, embedded bars, and externally bonded reinforcement (EBR) sheets in various combinations. This investigation includes 12 standardized beam samples to illustrate applying the proposed analysis procedure.

The verification capability of the deformation monitoring results defined the second condition for developing the testing setup shown in Figure 2a. This study employs three independent groups of monitoring devices of the pure-bending zone: two sets of linear variable displacement transducers (LVDTs) capture the vertical and longitudinal displacements, and a digital image correlation (DIC) system monitors the cracking process and surface deformations; the LVDTs and DIC system monitor deformations of the opposite sides denoted as “①” and “②” in Figure 2b. The LVDT devices *L*_10_–*L*_15_ estimate deformations of the side surface ①, and *L*_1_–*L*_3_, *L*_4_–*L*_6_, and *L*_7_–*L*_9_ indicators monitor the vertical displacements. The DIC captures the deformations and crack patterns on the side surface, designated as “②”. Figure 2b illustrates the DIC setup, employing two LaVision VC-Image E-Lite 5M cameras placed on a tripod 3.0 m from the exposition samples and 0.5 m apart. The cameras with charge-coupled device (CCD) detectors have a 2456 × 2085 pixel resolution and operate at 12.2 frames per second. The lighting equipment Arri ensures the quality and accuracy of the digital images.

Table 1 describes the beam specimens’ geometry and material properties. In this table, *h* and *b* represent the height and width of the cross-section in Figure 1a; *d*, *A*, and *E* are the reinforcement parameters (effective depth, cross-section area, and modulus of elasticity); *f_t_* describes the tensile strength of the reinforcement, and the subscripts “1” and “2” correspond to the transformed reinforcement components in Equation 1. Table 1 also shows the compressive strength of the standard ∅150 × 300 mm concrete cylinder (*f’_c_*) on the testing day; the column “Age” specifies the testing age. The column “*f’_c_*” specifies the average value and standard deviation determined for four identical cylinders; the remaining parameters of the concrete necessary for the analysis (i.e., the modulus of elasticity and tensile strength) were determined using the average compressive strength values (Table 1) and the Eurocode 2 formulas [44]. Gribniak et al. [45,46] proved the adequacy of such an approach for the numerical analysis of RC elements. The manufacturers reported mechanical properties and cross-section parameters of the polymeric reinforcement materials for EBR, NSM, and GFRP systems; the tensile tests of three identical samples [47] determined the characteristics of steel reinforcement listed in Table 1.

For comparison purposes of the composite reinforcement systems, Table 1 also includes the transformed modular ratio and reinforcement ratio product [34]:

(6) $$n\rho =\frac{E_{r}}{E_{c}}\cdot \frac{A_{r}}{bd_{r}}.$$

Figure 2b shows the loading setup. A servo-hydraulic testing machine LVF5000 with a 5 MN capacity (Walter + Bai ag., Löhningen, Switzerland) loads the test samples with a 0.4 mm/min velocity; Dion 7 software ensures the loading system control and data acquisition process. The load cycles are formed in a semi-automatic manner with manual load reduction to the minimum cycle boundary. The data logger Almemo 5690-2 collects the load cell and LVDT outputs every second.

**Figure 3:**
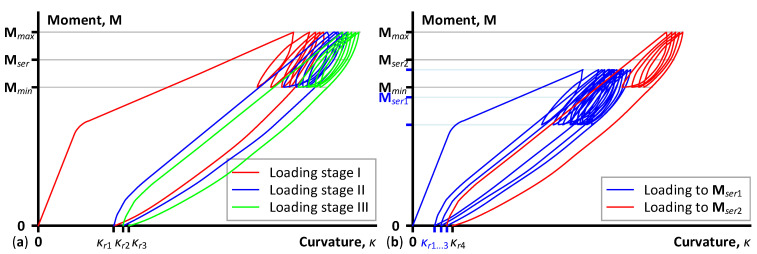
Loading protocols: (**a**) cycling loads over the service moment (load type “B”, Table 2); (**b**) different intensity loads (type “C”, Table 2).

This study focuses on the stiffness decrease under repeated mechanical loads. Still, the analytical model (Section 2.1) does not require load history specification—it explicitly relates the bending moment and curvature values in the pure-bending zone. Thus, in any combination, it remains equally efficient for high cycle and repeated loads, including temperature, creep, aggressive chemicals, and mechanical loads. The considered load cycles are essential for analyzing repeated factors when the mechanical load is necessary to estimate the residual stiffness of the beam sample, e.g., after the harsh environmental impacts. Figure 3 schematically depicts the loading application cycles—each loading stage consists of five load cycles with a 15% fluctuation about the target load referred to as the service moment, **M***_ser_*. These load cycling numbers were set arbitrarily in this study to avoid measurement errors and provide several data points for averaging.

During the physical tests, the loading process starts automatically at 0.4 mm/min until the maximum load of the cycle **M***_max_* (exceeding **M***_ser_* by 15%). Subsequently, the operator interrupts the loading process when it reaches the upper boundary **M***_max_*, manually reducing the load to the minimum cycle boundary **M***_min_* (corresponding to approximately 85% of **M***_ser_*). After that, the bending moment rises again under computer control to the maximum cycle load **M***_max_*, continuing the loading cycles. The complete unloading finalizes the first loading stage, resulting in the residual deformation expressed as curvature *κ_r_*_1_. Typically, this loading process is repeated three times (with five load cycles in each repetition) with the complete unloading of the beam samples, investigating the residual deformation trends. Each subsequent loading stage starts at the same speed as the previous loading round. However, the curvature analysis accounts for the residual deformations from all past loading stages (*κ_r_*_1_, *κ_r_*_2_, *κ_r_*_3_ …), shifting the curvature diagrams as shown in Figure 3. To determine the loading conditions in terms of the ultimate load **M***_ult_*, Table 2 uses the following notation:

- The sign “≡” relates the loading conditions to the particular beam sample. For instance, the symbol “≡S1” refers to the loading condition of the S1-EBR beam, determining the exact service moment (**M***_ser_*) and the cycle boundaries (**M***_min_* and **M***_max_*).
- Gribniak et al. [20] tested the identical element to the S6-GFRP sample until failure under monotonic load, which determines the loading conditions in this study.
- In the element with steel reinforcement (S12-S), the theoretical moment, corresponding to the steel yielding, limits the ultimate cycle load **M***_max_*; the service load **M***_ser_* was set to exceed the cracking moment calculated by Eurocode 2 formulas [44].
- The target loadings of the elements, combining steel and NSM reinforcements, were set to represent the loading conditions of alternative test samples (S1-EBR, S4-NSM, and S12-S) for comparison purposes.

Figure 3 schematically depicts the loading layouts “B” and “C” from Table 2. The monotonic tests (type “A”) or previous experimental program [20] determined the service load, representing approximately 55% of the load-bearing capacity of nominally identical samples, tested under monotonic load until failure (except for the S12-S sample, whose loading parameters are discussed above). This limitation came from the design principles of steel reinforcement when the efficient design of the bending sample ensures 50–60% stresses in the tension reinforcement regarding the yielding strength of the steel (e.g., [37]). Gribniak et al. [45] conducted a detailed statistical analysis of the service load conditions. However, the definition of FRP reinforcement systems’ service load is a more complex problem. The necessity to compare the deformation of the elements with steel and GFRP bars defines the typical analysis issue.

Gribniak et al. [46] proposed the analysis methodology, which compares the statistical data corresponding to the identical stresses in the tensile reinforcement. However, this investigation cannot employ the latter approach mainly because of these simplified laboratory samples’ limited dimensions and reinforcement detailing restrictions. Therefore, this study makes no difference between the test samples’ failure mechanisms determining the ultimate load. Independently of the reinforcement parameters, the service moment represents 55% of the load-bearing capacity of the identical beam sample tested under monotonic load. The limitations of this assumption are evident since a 30% magnitude also appears in the literature (e.g., [18,48]). However, any possible approaches still result in comparison subjectivity, and the efficiency analysis of the reinforcement should account for specific limitations in real projects.

### 2.3. Experimental Program

The illustrative experimental program employs laboratory-mixed concrete, using the same mix design as the previous studies [20,22,29]. The proportions for a cubic meter are the following: cement CEM I 42.5 R = 356 kg, water = 163 L, limestone powder = 177 kg, 0/4 mm sand = 890 kg, 4/16 mm crushed dolomite aggregates = 801 kg, superplasticizer Mapei Dynamon XTend = 1.97% (by the cement weight), and admixture SCP 1000 Optimiser = 3.5 kg. In addition, two types of synthetic fibers (0.9 kg of Crackstop M Ultra ∅0.022 × 13 mm and 4.2 kg of Durus EasyFinish ∅0.7 × 40 mm) were used in the concrete to avoid a sudden failure of the shear zone. All the beams were produced in steel forms and were unmolded 2–3 days after the casting. After that, all samples were stored in laboratory conditions at an average of 73% humidity and 20 °C before the testing day or forming external FRP reinforcement systems.

**Figure 4:**
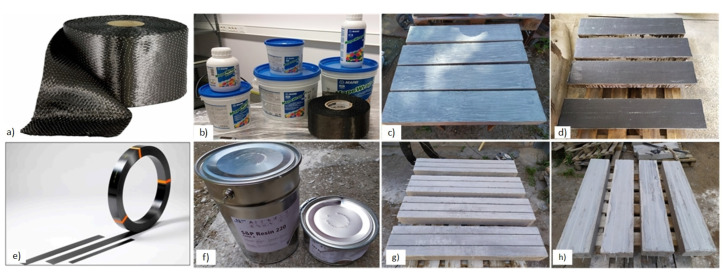
Installing reinforcement systems: (**a**) CF sheets; (**b**) epoxy adhesive; (**c**) prepared surface of specimens; (**d**) specimen with adhesively bonded CF sheets; (**e**) S&P C-Laminate CFRP strip; (**f**) S&P Resin 220 epoxy adhesive; (**g**) arrangement of the NSM grooves; (**h**) specimen with embedded CFRP strips. Note: the beam samples are shown in an inverted position to clarify the installation details.

As Table 1 shows, the test program includes four specimen types. In particular, the abbreviation EBR corresponds to externally bonded reinforcement using carbon fiber (CF) sheets. Thus, two unidirectional MapeWrap C UNI-AX sheets with the dry fabric’s 100 × 0.164 mm equivalent thickness were attached to the most tensioned surface of the beam samples. These sheets were placed along the beam and attached to the concrete surface using a two-component MapeWrap 31 epoxy resin (Figure 4a–d). Before the EBR bonding, the cleaned concrete surface was leveled with epoxy putty and primer. The adhesive was allowed to dry for seven days before conducting the tests.

The NSM abbreviation in Table 1 describes a near-surface-mounted reinforcement system. The notation S/NSM corresponds to the NSM system formed on the beam sample with two 5 mm embedded steel bars, creating a hybrid reinforcement system. The NSM component consisted of two pultruded 10 × 1.4 mm carbon fiber-reinforced polymer (CFRP) strips (S&P C-Laminate) installed in the 12 × 4 mm grooves milled at the bottom surface of the specimens (Figure 4e–h). Before placing the strips, the grooves were filled with a two-component S&P Resin 220 epoxy adhesive. Excess epoxy was removed with a spatula to ensure the test samples’ surface was even. The adhesive was allowed to cure for seven days before conducting the mechanical tests. Remarkably, the NSM system of the S12-S/NSM sample was formed after testing the S12-S beam with two 5 mm steel bars, simulating an RC structure strengthening consequence.

**Figure 5:**
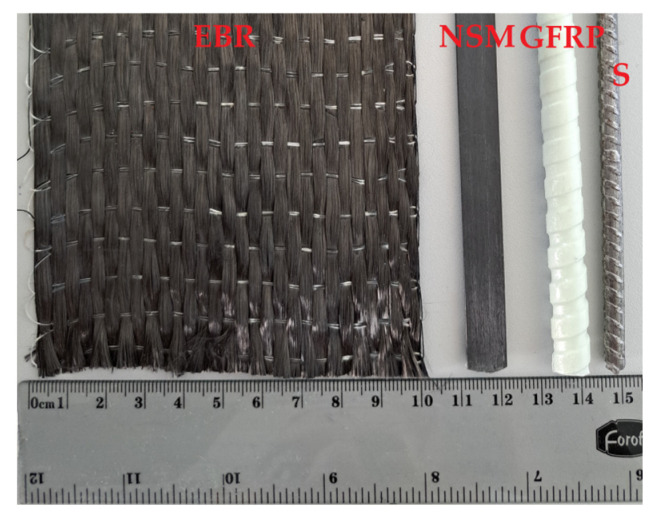
The surface treatment of the reinforcement materials.

This study also includes beams with two embedded 8 mm glass fiber-reinforced polymer (GFRP) bars. The GFRP reinforcement system employs ComBar bars from Shöck. Figure 5 shows the surface treatment of all the reinforcement materials in this work.

## 3. Results

**Figure 6:**
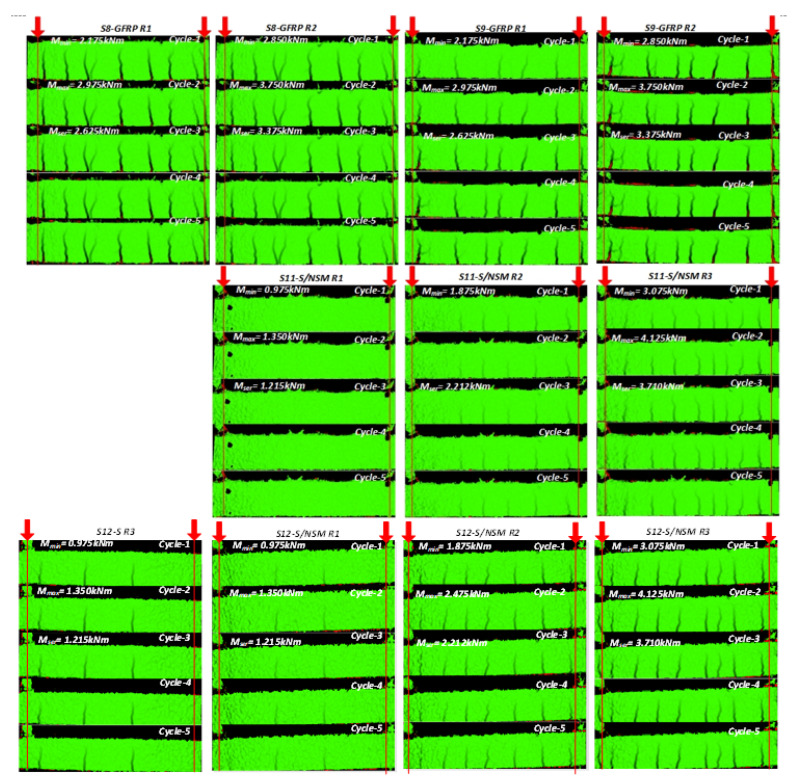
Cracking patterns of the selected samples identified by the DIC system. Note: the red arrows indicate the load application points.

Figure 6 demonstrates the crack propagation of several selected beam samples during the five load cycles corresponding to succeeding load repetitions (*R1*, *R2*, *R3*, etc.) captured with the DIC system. However, these cracking results could only qualitatively illustrate the composite performance of the reinforcement system in involving the tension concrete resisting the load. Thus, the number of cracks (in the post-cracking stage) could reveal the tension-stiffening effect [49]. Remarkably, the proposed setup produces multiple cracks in the 600 mm long pure-bending zone, which makes it suitable for the tension-stiffening analysis using the smeared crack model, e.g., as described in reference [38].

Gribniak et al. [22] also demonstrated the feasibility of the simplified analytical model (Section 2.1) for the residual strength analysis. Thus, the stiffness analysis employs monitoring results of the pure-bending zone. The indicator distribution scheme (Figure 2) ensures the curvature estimation using three independent measurement sets, i.e., the vertical displacements registered by the *L*_1_–*L*_9_ LVDT devices and the surface deformations captured by DIC and LVDTs. This instrumentation allows cross-verifying the test measurements, which is vital during the cyclic tests because of a certain inertness of the LVDT devices under reversed load and DIC sensitivity to the cameras’ movements [20,50]. Gribniak et al. [22] described the curvature analysis procedure in detail; the present study indicated that vertical displacements produce reliable curvature values, defining the analysis object. Following this approach, the averaged curvature over the pure-bending zone can be determined as follows:

(7) $$\begin{array}{cc} \kappa =8\Delta /\left(l^{2}+4\Delta^{2}\right), & \Delta =\left(u_{4}+u_{5}+u_{6}\right)/3 \end{array}-\left(u_{1}+u_{2}+u_{3}+u_{7}+u_{8}+u_{9}\right)/6,$$

where Δ is the deflection over the pure-bending zone having length *l* equal to 600 mm, as Figure 2a shows; *u_i_* is the displacement obtained by the *L_i_* device (*i* = 1…9).

**Figure 7:**
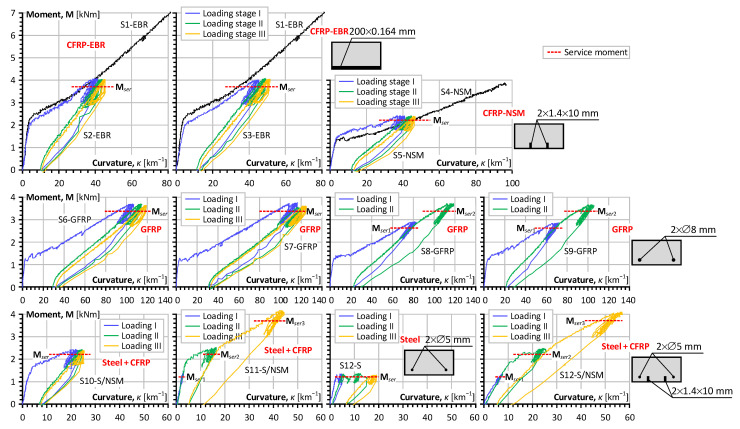
Moment–curvature results of the test samples from Table 1.

Figure 7 shows the corresponding moment–curvature diagrams of all beam samples from Table 1 and Table 2. The sub-charts in this figure have identical ordinate scales, but the abscissa scales in each row differ. This figure also indicates the service moments for the residual stiffness comparison. However, this analysis is barely possible straightforwardly because of the differences in the modulus of elasticity of reinforcement and geometry characteristics (Table 1). Therefore, this study uses the equivalent stress ($\sigma_{t}^{*}$) to measure and compare the stiffness decay with the load.

Remarkably, complete reloading followed all load cycling sets to measure the residual deformation of the beam samples. The latter values are vital for the residual stiffness analysis—the origin of each consequent diagram (Figure 3 and Figure 7) coincides with the reloading deformation (residual curvature) from the preceding loading stage, producing the adequate resultant curvature suitable for Equation (5).

Table 3 shows the residual deformations (curvatures) determined for all samples subjected to unloading repetitions. These values are mandatory for adequate analysis when the mechanical load, necessary to estimate the residual stiffness of the beam sample, describes the consequential shift of the moment–curvature diagrams after reloading cycles (Figure 3). Thus, Table 3 determines the essential outcome valuable for further analysis and the modeling of the composite reinforcement systems similar to those considered in this work.

## 4. Discussion

### 4.1. The Cyclic Load Effect

The residual curvature analysis of the results from Table 3 reveals similar resultant deformations of all beam samples (except for elements with GFRP reinforcement), which were almost independent of the *nρ* ratio (Table 1) and loading layout and intensity (Figure 7). In addition, the steel-reinforced sample (S12-S) under the 1.215 kNm service moment demonstrates the 9.4 km^−1^ residual curvature comparable to the 11.2 km^−1^ result of the same element after strengthening (S12-S/NSM) subjected to 3.710 kNm load. This outcome demonstrates the efficiency of the strengthening solution.

Increasing the sample numbers will help identify more meaningful tendencies. However, Table 3 already shows the exceptional vulnerability of GFRP reinforcement systems to repeated load. For example, the S6 to S9 beams demonstrate tripled residual curvatures regarding the elements with EBR sheets, and hybrid reinforcement systems reached higher bending moments comparing the GFRP counterparts. Still, the S11-S/NSM and S12-S/NSM samples had a doubled *nρ* value, i.e., 2.45% vs. 1.22% (Table 1), which could explain the residual resistance increase. However, the EBR elements had a similar *nρ* ratio (1.18%). This allows the authors to hypothesize that the embedded GFRP bars cannot ensure sufficient resistance to repeated loads because of high deformability, corrupting the bond with concrete. The following equivalent stiffness analysis checks this hypothesis.

**Figure 8:**
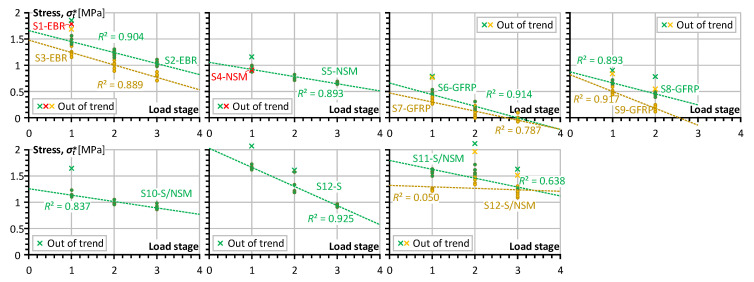
The decrease in the equivalent tensile stress ($\sigma_{t}^{*}$) with loading. Note: the red color corresponds to the samples subjected to monotonic load; the yellow and green colors show the test results of alternative counterparts.

The moment–curvature diagrams in Figure 7 produce the initial data for the analysis, and Equation (5) determines the equivalent stresses ($\sigma_{t}^{*}$) corresponding to the service load. Thus, Figure 8 shows the residual stress diagrams corresponding to the load cycles, grouped by the reinforcement type and loading layout. Therefore, analyzing the charts in Figure 8 requires referring to Table 2, which indicates the loading conditions for every load stage.

**Figure 9:**
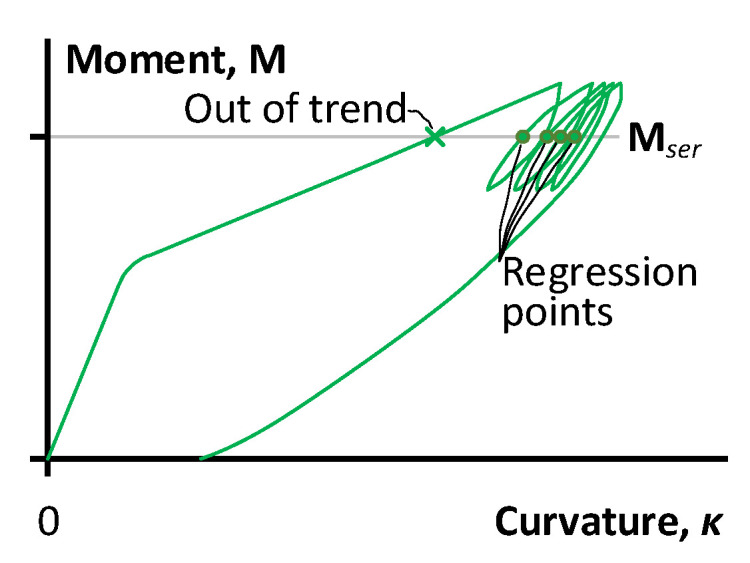
Schematic identification of the moment–curvature points for regression analysis in Figure 8.

Figure 9 schematically depicts the curvature estimation procedure, where only ascending loading diagram branches produce the analysis points. This sketch represents a smoothed view of the experimental diagram and, thus, is a reliable illustrative example. The analysis of the regression trends in Figure 8 reveals the essential differences between the refused points, and the remaining points belong to the first loading cycle. The further load repetitions do not include such outliers (except for the S12 sample after strengthening).

The schematic in Figure 9 illustrates the situation when the first branch of the moment–curvature diagram does not generate the data point for determining the trend line of the stress degradation because of the essential difference in the element stiffness before and after the load cycles. The inclusion of such exceptional results will corrupt the stiffness trends. Therefore, only four data points (except for the first loading point) are employed for the residual strength regression analysis.

### 4.2. The Reinforcement Effect

In addition to the tripled residual curvatures (Table 3), Figure 8 demonstrates the minimal efficiency of GFRP bars in terms of the equivalent tensile stresses, which describes the composite behavior of reinforced concrete [20,22,29]. In particular, the S6-GFRP and S7-GFRP elements, which were subjected to cycling load with a 3.375 kNm service moment, demonstrate the complete disappearance of the tensile stresses in the concrete. A less eager loading in the S8-GFRP and S9-GFRP beams (Table 2) explains an improvement of equivalent stress values estimated at the first loading cycle (Figure 8). However, the identified tendency indicates the concrete contribution disappearing corresponding to the third loading stage. The mechanical bond degrading in concrete because of a relatively low stiffness of the GFRP bars describes the possible explanation for this effect. This observation aligns with the literature results (e.g., [12,13]). Unfortunately, such experimental investigations are rare in the literature; most such experimental works consider the pull-out behavior of GFRP bars. At the same time, the bonding problem of the embedded GFRP bars becomes apparent only for repeated load situations—Gribniak et al. [22] did not identify the bond deterioration problems of GFRP bars under monotonic load for the same reinforcement configuration. On the other hand, this drawback is untypical for alternative reinforcement schemes, making them acceptable to replace embedded GFRP bars.

A sharp inclination of the trend line of the equivalent stresses of the S12-S sample (with only steel reinforcement) highlights another bond-degrading mechanism characteristic of embedded bars. This stiffness decrease results from the cover cracking and the corresponding reduction in the concrete efficiency—only the concrete between the bar reinforcement and compressive zone (Figure 1b) contributes to the mechanical resistance of the cross-section. This observation aligns with the previous test findings [20,22,29], but the load cycles made this degradation mechanism more transparent. Although the load intensity of the S12-S beam was relatively high, i.e., the service load represents 85% of the theoretical moment of the steel yielding (Table 2), this load is well below the service loads faced by the remaining test samples (Table 3). On the other hand, this element possesses the highest *nρ* ratio (1.83%) among the test samples (except for the hybrid reinforcement systems). This outcome proclaims the inefficiency of the typical steel reinforcement for resisting cycling load, requiring a further unpractical *nρ* ratio increase. Furthermore, it reveals the need to renew structural design principles and tailor the materials’ mechanical properties for construction [51]. This analysis procedure opposes the current practice, associating standardized engineering solutions with existing materials, the physical characteristics of which are imperfectly suiting the structural requirements and leading to an inefficient increase in the material amount for safety’s sake.

In this context, the CFRP reinforcement systems demonstrate outstanding performance under the cycling load. The equivalent stresses in the S1 to S3 EBR samples reach the 1.86 MPa value and do not decrease below 0.70 MPa under the 3.71 kNm service load cycling; the NSM systems demonstrate the 1.16 MPa stress and preserve the minimum 0.64 MPa stresses, though these values correspond to the 2.22 kNm bending moment. The S10-S/NSM hybrid system improves the latter values correspondingly to 1.65 MPa and 0.87 MPa with the same tendency of stress decay as the S5-NSM beam. Under the increased load cycles, the S11-S/NSM proves a further increase in mechanical performance: the maximum and minimum equivalent stresses are equal to 2.11 MPa and 1.15 MPa.

The S12-S/NSM beam represents the strengthening situation of the S12-S element. At the load stages “2” and “3”, the S12-S/NSM sample demonstrates very similar equivalent stresses to the S11-S/NSM beam (Figure 8). However, the identified trend line reveals a remarkable tendency—the loading cycles do not affect the residual resistance of the strengthened sample, preserving the averaged equivalent stresses at an approximately 1.3 MPa level. This finding supports the efficiency of the NSM strengthening systems for the mechanical load cycles. Further studies should reveal the hybrid reinforcement system’s efficient layout and steel-to-CFRP proportions.

**Figure 10:**
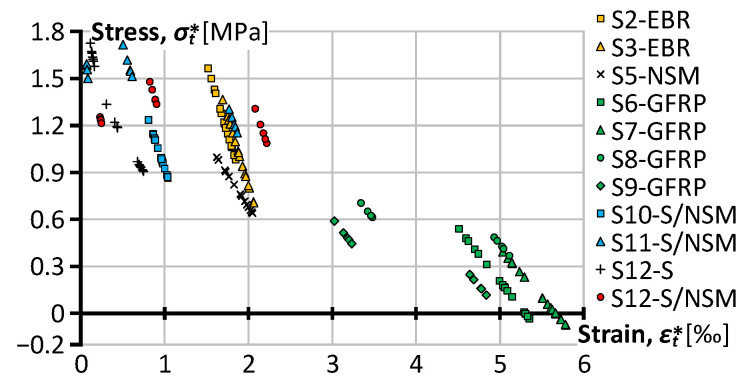
The equivalent stress–strain values correspond to the results of Figure 8.

Figure 10 illustrates the results by relating the equivalent stress and strain values, i.e., Equation (5). This figure, including only “regression points” from Figure 8, demonstrates the stress decrease tendency with strain. This tendency is apparent for the GFRP beam samples (green-filled markers). Thus, seemingly, the relatively low modulus of elasticity of GFRP bars (Table 1) increases the concrete strains, causing a loss of the bond performance. The remaining reinforcement systems limit the deformations, which do not exceed a third of GFRP values. However, only the hybrid systems (S11-S/NSM and S12-S/NSM) prevent the reduction in the equivalent stresses under the load repetitions, ensuring reinforcement efficiency.

### 4.3. The Load Intensity Effect

The results of Figure 8 and Figure 10 demonstrate the essential importance of the load intensity on the residual stiffness decay. In particular, this effect is apparent in the S12-S beam sample subjected to 85% of the maximum theoretical load. The sharp decrease in the equivalent stresses results from this almost ultimate behavior.

Table 4 summarizes the results of Figure 10 in average terms. In addition, this table estimates the alteration of the equivalent stresses as a function of the equivalent strains. The following expression determines the stiffness alteration ratio:

(8) $$\begin{array}{cc} \frac{{∆\sigma }_{t}^{*}}{∆\varepsilon_{t}^{*}}=\frac{\sigma_{t,i}^{*}\;-\;\sigma_{t,i-1}^{*}}{\varepsilon_{t,i}^{*}\;-\;\varepsilon_{t,i-1}^{*}}, & i=2,\;3 \end{array}.$$

Here, the subscript *i* describes the values corresponding to successive loading stages; the negative ratio corresponds to the average stress reduction because of the load repetitions.

In addition to the beam S12-S discussed above, Table 4 reveals the most substantial decrease in the residual stiffness of the EBR (S2 and S3) samples expressed in the ratio ${∆\sigma }_{t}^{*}/∆\varepsilon_{t}^{*}$. A relatively high service moment (Table 2) in combination with a relatively low resistance of CF sheets to transverse (shear) load [20] could explain the intensive stress decrease. At the same time, these specimens still demonstrate substantial residual stiffness expressed in the equivalent stresses’ terms because of the significant bonding area of EBR (Figure 4h).

Comparing the stiffness decay tendency (Table 4) of the S10-S/NSM and S11-S/NSM samples reveals a surprising outcome related to the positive correlation between the equivalent stresses and the service load (${∆\sigma }_{t}^{*}/∆\varepsilon_{t}^{*}>0$) of the S11 specimen. Analysis of Figure 7 can explain this issue—the service load of the S10 beam sample belongs to the crack formation stage, which predominantly controls the stiffness decrease. Analyzing the mechanical response of the S6 to S9 GFRP-reinforced beams provides the opposite case when the deformations exceed the concrete bonding limit, making the reinforcement inefficient ($\sigma_{t}^{*}\approx 0$). However, this work only exemplifies the proposed residual stiffness analysis procedure. Further studies should consider the load intensity effect and form the corresponding testing protocols.

## 5. Conclusions

This manuscript proposes composite reinforcement systems’ residual stiffness analysis procedure under repeated mechanical loads. The experimental program demonstrates the effectiveness of the proposed methodology, analyzing the bending test results of 12 beam samples with various reinforcement types. The following essential conclusions result from this work:

- The proposed testing procedure is suitable for quantifying the residual stiffness decrease under repeated mechanical load, including the complete load removal between the loading cycles. The quantification employs the equivalent stresses acting in the concrete in tension under the assumption of the rectangular stress distribution. This simplified model approximates the tensile stresses with sufficient accuracy—the average approximation error (regarding the “exact” solution) does not exceed 7%. On the other hand, the “exact” inverse analysis reported in the literature is inapplicable to the specimens under cycling loads, which makes the proposed methodology irreplaceable for this study’s purpose.
- This study reveals a limited ability of glass fiber-reinforced polymer (GFRP) bars to ensure the bonding performance under repeated loads. The concrete deformations exceed the bonding limit, making the reinforcement inefficient because of a relatively low modulus of elasticity (60 GPa) of the GFRP bars. Moreover, the bonding problem becomes apparent only for repeated loads—the previous tests did not identify the bond deterioration problems of GFRP bars under monotonic load for the same reinforcement configurations. On the other hand, this drawback is untypical for alternative reinforcement schemes considered in this study, proving the viability of the proposed analysis methodology.
- The carbon fiber (CF) reinforced materials demonstrate outstanding mechanical performance under repeated loads. The externally bonded reinforcement (EBR) system ensures the equivalent stresses, which do not decrease below 0.70 MPa; the near-surface mounted (NSM) system preserves the minimum 0.64 MPa stresses. The hybrid reinforcement system, combining steel bars and NSM CFRP strips, improves this value to 0.87 MPa. Under the increased load cycles, the hybrid reinforcement demonstrates a further increase in mechanical performance—the equivalent stresses exceed 1.15 MPa, exceeding 50% of the tensile resistance of the concrete.
- The NSM reinforcement system efficiently strengthened the beam sample with steel reinforcement bars tested until 85% of the theoretical load-bearing capacity. The load repetitions did not affect the residual resistance of the strengthened specimen, preserving the averaged equivalent stresses at an approximately 1.3 MPa level. Further studies should reveal the hybrid reinforcement system’s efficient layout and steel-to-CFRP ratio.

## Figures and Tables

**Table 1:** Geometry and material parameters of the beam samples.

Beam	*h*	*b*	*d*_1_	*d*_2_	*A*_1_	*A*_2_	*E*_1_	*E*_2_	*f_t_*_,1_	*f_t_*_,2_	*nρ*	*f’_c_*	Age
	(mm)				(mm^2^)		(GPa)		(MPa)		(%)	(MPa)	(Days)
S1-EBR	103	199	104	–	32.8	–	230	–	4830	–	1.03	50.69 ± 1.53	47
S2-EBR	104	198	105	–	32.8	–	230	–	4830	–	1.13	35.12 ± 2.63	22
S3-EBR	102	198	103	–	32.8	–	230	–	4830	–	1.18	32.98 ± 3.15	21
S4-NSM	100	201	90	–	28.0	–	170	–	2800	–	0.83	34.12 ± 2.48	21
S5-NSM	106	201	96	–	28.0	–	170	–	2800	–	0.78	34.62 ± 2.92	21
S6-GFRP	104	200	77	–	100.6	–	60	–	1490	–	1.22	35.12 ± 2.61	22
S7-GFRP	106	197	76	–	100.6	–	60	–	1490	–	1.22	34.62 ± 2.48	21
S8-GFRP	102	202	74	–	100.6	–	60	–	1490	–	1.11	40.80 ± 1.63	13
S9-GFRP	107	201	81	–	100.6	–	60	–	1490	–	1.26	40.80 ± 1.63	13
S10-S/NSM	107	200	72	97	38.11	28	206	170	503.9	2800	2.45	32.98 ± 3.15	22
S11-S/NSM	108	197	77	92	38.11	28	206	170	503.9	2800	2.30	40.80 ± 1.63	14
S12-S	101	200	67	–	38.11	–	206	–	503.9	–	1.83	34.12 ± 2.92	19
S12-S/NSM				92		28		170		2800	2.45	35.23 ± 3.05	26

**Table 2:** Loading parameters of the beam samples (kNm).

Beam	Load Type	M*_min_*	M*_max_*	M*_ser_*	M*_ult_*
S1-EBR	A	–	–	3.710	7.035
S2-EBR	B	3.075	4.125	3.710	≡S1(*)
S3-EBR	B	3.075	4.125	3.710	≡S1
S4-NSM	A	–	–	2.220	4.005
S5-NSM	B	1.875	2.475	2.220	≡S4
S6-GFRP	B	2.850	3.750	3.375	6.527(†)
S7-GFRP	B	2.850	3.750	3.375	≡S6
S8-GFRP	C	2.175	2.975	2.625	≡S6
		2.850	3.750	3.375	
S9-GFRP	C	2.175	2.975	2.625	≡S6
		2.850	3.750	3.375	
S10-S/NSM	B	1.875	2.475	2.220	≡S4
S11-S/NSM	C	0.975	1.350	1.215	1.42(‡)
		1.875	2.475	2.220	≡S4
		3.075	4.125	3.710	≡S1
S12-S	B	0.975	1.350	1.215	1.42(‡)
S12-S/NSM	C	0.975	1.350	1.215	1.42(‡)
		1.875	2.475	2.212	≡S4
		3.075	4.125	3.710	≡S1

Note: (*) the sign “≡” means “equivalent to”; (†) Gribniak et al. [20] tested the identical element until failure under monotonic load; (‡) the theoretical moment corresponding to the steel yielding limits the upper cycle boundary.

**Table 3:** Residual curvatures (*κ_res_*) of the beams subjected to repeated loadings.

Beam	Load Stage I		Load Stage II		Load Stage III		Total Result	
	M*_ser_* (kNm)	*κ_res_* (km^−1^)	M*_ser_* (kNm)	*κ_res_* (km^−1^)	M*_ser_* (kNm)	*κ_res_* (km^−1^)	M*_max_* (kNm)	Σ*κ_res_* (km^−1^)
S2-EBR	3.710	9.893	3.710	0.719	3.710	0.500	4.125	11.11
S3-EBR	3.710	11.23	3.710	1.448	3.710	0.863	4.125	13.54
S5-NSM	2.220	11.75	2.220	1.285	2.220	1.059	2.475	14.10
S6-GFRP	3.375	29.08	3.375	3.052	3.375	3.989	3.750	36.12
S7-GFRP	3.375	30.88	3.375	2.159	3.375	4.274	3.750	37.32
S8-GFRP	2.625	22.64	3.375	6.693	–	–	3.750	29.33
S9-GFRP	2.625	21.32	3.375	6.819	–	–	3.750	28.14
S10-S/NSM	2.212	7.084	2.212	0.522	2.212	1.937	2.475	9.544
S11-S/NSM	1.215	0.548	2.212	4.585	3.710	5.859	4.125	10.99
S12-S	1.215	1.100	1.215	5.570	1.215	2.767	1.350	9.437
S12-S/NSM	1.215	0.604	2.212	4.856	3.710	5.744	4.125	11.20

**Table 4:** The average equivalent stresses and strains and the stiffness alteration ratio in Figure 10.

Beam	Load Stage I		Load Stage II			Load Stage III		
	$\sigma_{t}^{*}$ (MPa)	$\varepsilon_{t}^{*}$ (‰)	$\sigma_{t}^{*}$ (MPa)	$\varepsilon_{t}^{*}$ (‰)	${∆\sigma }_{t}^{*}/∆\varepsilon_{t}^{*}$ (GPa)	$\sigma_{t}^{*}$ (MPa)	$\varepsilon_{t}^{*}$ (‰)	${∆\sigma }_{t}^{*}/∆\varepsilon_{t}^{*}$ (GPa)
S2-EBR	1.474	1.570	1.224	1.710	−1.787	1.040	1.815	−1.757
S3-EBR	1.268	1.749	1.000	1.899	−1.791	0.771	2.029	−1.763
S5-NSM	0.949	1.679	0.761	1.903	−0.838	0.660	2.026	−0.825
S6-GFRP	0.472	4.610	0.196	5.014	−0.683	0.009	5.290	−0.680
S7-GFRP	0.348	5.107	0.075	5.546	−0.621	−0.021	5.700	−0.619
S8-GFRP	0.655	3.418	0.431	5.018	−0.140	–	–	–
S9-GFRP	0.514	3.137	0.180	4.743	−0.208	–	–	–
S10-S/NSM	1.160	0.857	0.990	0.963	−1.613	0.902	1.018	−1.581
S11-S/NSM	1.573	0.073	1.590	0.572	0.033	1.222	1.824	−0.294
S12-S	1.674	0.131	1.654	0.318	−1.712	0.937	0.715	−1.050
S12-S/NSM	1.240	0.239	1.394	0.873	0.243	1.173	2.166	−0.171

## Data Availability

The authors will provide the raw data of this work upon request.
